# Neurodevelopmental outcomes of preterm with necrotizing enterocolitis: a systematic review and meta-analysis

**DOI:** 10.1007/s00431-024-05569-5

**Published:** 2024-04-30

**Authors:** Yan Wang, Shunli Liu, Meizhu Lu, Tao Huang, Lan Huang

**Affiliations:** 1 Department of Emergency, West China Second University Hospital, Sichuan University, Chengdu, China; 2 Department of Pediatrics, West China Second University Hospital, Sichuan University, Chengdu, China; 3 Key Laboratory of Birth Defects and Related Diseases of Women and Children (Sichuan University), Ministry of Education, Chengdu, China

**Keywords:** Necrotizing enterocolitis, Preterm, Brain injury, Neurodevelopmental impairment

## Abstract

While neonatal necrotising enterocolitis (NEC) is associated with high mortality rates in newborns, survivors can face long-term sequelae. However, the relationship between NEC and neurodevelopmental impairment (NDI) in preterm infants remains unclear. To explore the relationship between neonatal NEC and neurodevelopmental outcomes in preterm infants, we searched PubMed, EMBASE, and the Cochrane Library from their inception to February 2024 for relevant studies. Studies included were cohort or case–control studies reporting neurodevelopmental outcomes of NEC in preterm infants. Two independent investigators extracted data regarding brain damage and neurodevelopmental outcomes in these infants at a corrected age exceeding 12 months. Odds ratios (ORs) were pooled using a random effects model. We included 15 cohort studies and 18 case–control studies, encompassing 60,346 infants. Meta-analysis of unadjusted and adjusted ORs demonstrated a significant association between NEC and increased odds of NDI (OR 2.15, 95% CI 1.9–2.44; aOR 1.89, 95% CI 1.46–2.46). Regarding brain injury, pooled crude ORs indicated an association of NEC with severe intraventricular haemorrhage (IVH) (OR 1.42, 95% CI 1.06–1.92) and periventricular leucomalacia (PVL) (OR 2.55, 95% CI 1.76–3.69). When compared with conservatively treated NEC, surgical NEC potentially carries a higher risk of NDI (OR 1.78, 95% CI 1.09–2.93) and severe IVH (OR 1.57, 95% CI 1.20–2.06). However, the risk of PVL did not show a significant difference (OR 1.60, 95% CI 0.47–5.40).

*Conclusions*: Our meta-analysis provides evidence suggesting an association between NEC and NDI. Additionally, the severity of intestinal lesions appears to correlate with a higher risk of NDI. Further high-quality studies with comprehensive adjustments for potential confounding factors are required to definitively establish whether the association with NDI is causal.

**Table:** 

**What is Known:** *• NEC is a serious intestinal disease in the neonatal period with a high mortality rate, and surviving children may have digestive system sequelae.* *• Compared with non-NEC preterm infants, the reported incidences of brain injury and neurodevelopmental disorders in NEC preterm infants are not the same.*	
**What is New:** *• The risk of neonatal brain injury and neurodevelopmental disorders in preterm infants with NEC is higher than that in non-NEC infants, and the risk of NDI in surgical NEC infants is higher than that in the conservative treatment group.* *• NEC may increase the risk of motor, cognitive, language development delays, and attention deficits in children.*

**Supplementary Information:**

The online version contains supplementary material available at 10.1007/s00431-024-05569-5.

## Introduction

Necrotising enterocolitis (NEC) is a severe gastrointestinal disease that occurs during the neonatal period and is a major cause of neonatal mortality. Neonatal intensive care units report an incidence of 2–5%. This is particularly concerning for very low birth weight (VLBW) infants, who experience a 2–7% incidence rate [1]. Mortality rates for NEC remain high, with estimates reaching 20–30% [2]. Moreover, 9–36% of surviving children face long-term digestive system sequelae such as short bowel syndrome and intestinal stenosis [3]. Studies indicate a substantial financial burden on families and society due to the increased medical costs associated with NEC, especially for those requiring surgery during their early childhood development [4].

Beyond digestive system complications, children with NEC are at risk of neurodevelopmental impairment (NDI) [5]. The incidence of NDI in these children is approximately 40%, which is twice that in children without NEC [6]. Research suggests a higher risk of intracranial haemorrhage (IVH), periventricular leukomalacia (PVL), cerebral palsy (CP), and severe visual and hearing impairment in children with NEC. Additionally, compared with medical management, surgical intervention for NEC appears to elevate the risk of NDI. However, some studies have failed to find an increased risk of NDI in children with NEC during the neonatal period [4]. Although different types of NDIs in NEC survivors have been reported, most studies have not adjusted for other factors associated with brain injury, such as gestational age, birth weight, and infection. Notably, some studies suggest a higher risk of behavioural problems, such as attention deficits in NEC survivors during childhood [6], while others have not observed such a connection [7]. Our study aims to address these inconsistencies. We conducted a comprehensive review of NEC and its neurological sequelae in children. This analysis extends to systematically evaluate the risk of various functional and behavioural brain disorders in children with NEC. This information will serve as a valuable reference for targeted early rehabilitation interventions for children with NEC.

### Methods

Following the Preferred Reporting Items for Systematic Reviews and Meta-Analyses (PRISMA) guidelines [8], we conducted a systematic review and meta-analysis. The protocol and search strategies were registered in PROSPERO (CRD42024509168) (crd.york.ac.uk/PROSPERO/display_record.php?RecordID = 509,168).

### Retrieval of studies

A systematic search of PubMed, EMBASE, and the Cochrane Library was conducted to identify related studies. The search strategy used in this study is described in Supplementary data 1. Only human studies published in English were included in this analysis. When studies shared overlapping participants, the one with the largest sample size was chosen. We also identified additional candidates by manually searching the references of included research articles, meta-analyses, and reviews.

### Inclusion and exclusion criteria

Inclusion criteria for our study were as follows: (1) those with a cohort or case–control design; (2) those that reported brain injury IVH or PVL, neurodevelopmental outcomes CP, any disability, severe disability, visual impairment, hearing impairment, and language delay or behavioural difficulties (attention-deficit/hyperactivity disorder [ADHD] or autism) in preterm infants; (3) those with developmental follow-up performed at ≥ 1 year corrected age; (4) those with defined NEC diagnosis criteria (Bell’s stage II or III, or diagnosed clinically/radiologically); and (5) those that provided total participants, number of cases, odds ratios (ORs), hazard ratios (HRs), or risk ratios (RRs) with 95% confidence interval (CI).

Studies were excluded if they (1) were case reports, reviews, and animal studies; (2) focussed on term neonates; (3) had overlapping data; and (4) lacked raw data. Surgical NEC was defined as NEC requiring laparotomy, laparoscopy, or peritoneal drainage. Medical NECs were treated without surgical intervention.

### Data extraction and quality assessment

Two independent reviewers extracted data on the first author, publication year, country, study design, gestational age/birth weight, number of cases and total sample population, NDI category, age at follow-up, and developmental assessment scale. Differences in opinion were resolved through discussion among all reviewers.

The methodological quality of each study was examined using the Newcastle–Ottawa Scale (NOS) with a maximum score of nine. The quality of the studies was divided into three categories: high (score 7–9), moderate (score 4–6), and low (score 0–3) [9].

### Statistical analysis

Stata version 18.0 (Stata Corporation, College Station, TX, USA) was used for the meta-analysis. A random-effects model was employed as anticipated. Pooled estimates included both adjusted and unadjusted ORs with 95% CIs from the included studies. If studies lacked ORs, they were calculated from raw data. The *I*^2^ statistic (significance level, > 50%) and *Q* statistic (significance level, *P* < 0.10) were used to assess the heterogeneity between studies. We also performed a sensitivity analysis by sequentially excluding each study. The possibility of publication bias was visually assessed using Egger’s and Begg’s tests (significance level, *P* < 0.05). Additionally, the “trim and fill” procedure was performed to evaluate potential publication bias in our meta-analysis further.

## Results

### Literature search and selection

**Fig. 1:**
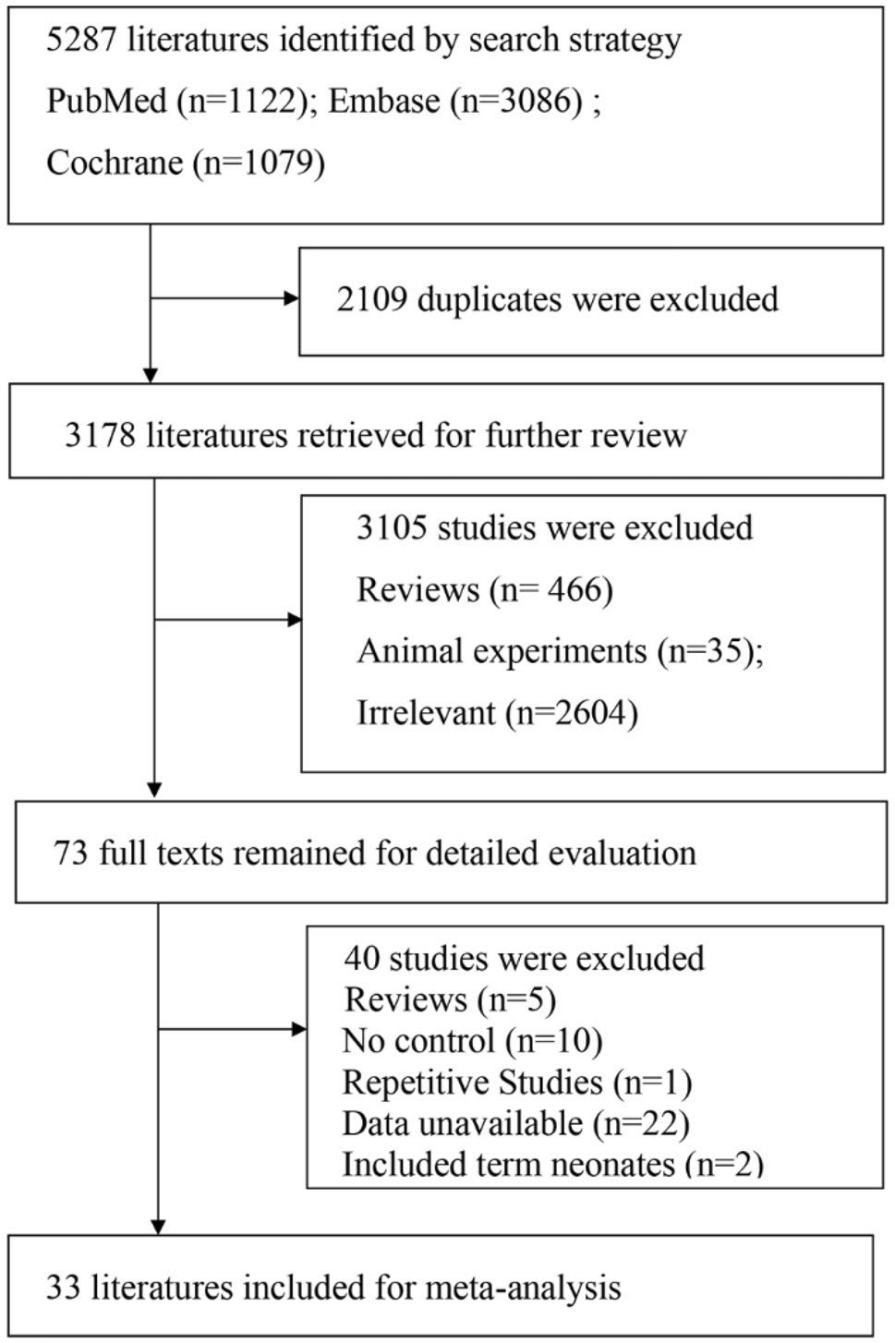
Flow diagram of the study selection process

A comprehensive search identified 5287 articles (PubMed, 1122; EMBASE, 3086; Cochrane Library, 1079). Following careful screening, 33 studies were selected for inclusion (Fig. 1). There were 15 cohort studies and 18 case–control studies.

### Study characteristics

The characteristics of the 33 selected studies are presented in Table 1. The selected studies spanned publication years between 1989 and 2023. Geographically, the studies originated from North America (14) [7, 10–22], Europe (9) [23–30], Australia (4) [31–34], China (1) [35], Japan (2) [36, 37], Taiwan (2) [38, 39], and one multicentre study [40]. Birth weight and gestational age criteria varied: 15 studies included infants < 1000 g or < 28 weeks gestation, 16 included those < 1500 g, and two studies involved all preterm infants. Per the NOS, the methodological quality was generally good, with 21 high-quality and 12 moderate-quality studies (Supplementary data 2 sTable 1).

**Table 1:** Characteristics of the studies included in the meta-analysis

**Study**	**Location**	**Design**	**NDI category**	**Total number**	**Gestational age/birth weight**	**Age at follow-up**	**Ascertainment of developmental impair**	**Study period of birth date**
Walsh [10]	USA	C	Baley mental score < 80 Abnormal neurosensory	802	VLBW	20 m	Bayley	1975.01–1983.12
Simon [11]	USA	CC	Neurodevelopment delay	18	VLBW	15 m	INFANIB DDST Bayley	1986–1988
Mayr [31]	Australia	CC	Hearing impairment	18	VLBW	1–12 y	N/A	1978–1991
Tobiansky [32]	Australia	CC	IVH Deaf Blind CP Mental delay	89	VLBW	12 m and 36 m	Griffiths Mental Development Scales	1986.01–1991.12
Waugh [33]	Australia	C	IVH CP Intellectual impairment	198	ELBW	24 m	Griffith General Quotient	1977.07–1990.02
Chacko [34]	Australia	CC	PVL IVH Developmental delay	40	ELBW	1–7 y	Griffith General Quotient Standford-Binet	1990–1993
Sonntag [23]	Germany	CC	Psychomotor retardation	60	VLBW	20 m	Griffith Developmental Scales	1992.01–1996.12
Salhab [13]	USA	CC	IVH Developmental delay	68	ELBW	18–22 m	BSID-II	1995–1998
Yeh [39]	Taiwan	CC	IVH PVL Developmental delay	45	VLBW	18 m	BSID-II	1991.01–2002.04
Hintz [12]	USA	C	IVH PVL CP Deaf Blind Developmental delay	2935	ELBW	18–22 m	BSID-II	1995.01–1998.12
Jen [14]	USA	CC	IVH	98	24–27 w /ELBW	N/A	N/A	1991.10–2003.12
Soraisham [22]	Canada	CC	IVH 3/4 PVL CP Deaf Blind Developmental delay	163	GW ≤ 32 w/ ≤ 1250 g	36 m	N/A	1995.01–2000.12
Bassler [40]	Canada, USA, Australia, New Zealand, and Hong Kong	C	CP Deaf Blind Developmental delay	944	ELBW	18 m	BSID-II	1996.01–1998.03
Lodha [37]	Japan	CC	Developmental delay	23	24–37 w	24–28 m	BSID-II	1997–1998
Martin [15]	USA	C	CP Developmental delay	1155	23–27 w	24 m	BSID-II, GMFCS	2002–2004
Saldir [24]	Turkey	C	Developmental delay	169	≤ 32 weeks	10–42 m	BSID-II	2002.01–2006.03
Maitre [16]	USA	CC	CP Developmental delay	60	VLBW	18 and 36 m	BSID-II, GMFCS	1998.01–2004.12
Roze [25]	Netherlands	CC	Severe cerebral pathology Behaviour difficulties	63	27–34 w	6–13 y	GMFCS WISC-III-NL	1996–2002
Pike [41]	UK	C	Functional impairment ADHD CP	6304	< 37 w	7 y	SDQ	1994.07–2000.08
Dilli [26]	Turkey	CC	CP Developmental delay	60	ELBW	18–24 m	BSID-II GMFCS	2007.10–2009.04
Shah [17]	USA	C	IVH PVL Developmental delay	1667	ELBW	18–22 m	BSID-II	1998.01–2009.07
Wadhawan [18]	USA	C	IVH PVL CP Blind Deaf Developmental delay	8656	ELBW	18–22 m	BSID-II	2000–2005
Hayakawa [36]	Japan	CC	Developmental delay	205	VLBW	18 m	N/A	2003.01–2012.12
Fullerton [20]	USA	C	Severe IVH or cystic PVL Blindness Hearing Impairment Cerebral Palsy	9929	ELBW	18 and 24 m	BSID-II or BSID-III	1999–2012
Allendorf [27]	Germany	CC	Developmental delay	76	VLBW	24 m	BSID-II	2006–2013
Humberg [28]	Germany	C	PVL CP Developmental delay	8002	VLBW	5–6 y	GMFCS WPPSI I-III	2009–2014
Zozaya [21]	Canada	C	IVH Developmental delay	1980	22–28^+6^w/ELBW	18–30 m	Bayley-III GMFCS	2010.01–2011.09
Chen [35]	China	CC	IVH Developmental delay	28	< 32 w	12–18 m	Gesell Developmental Schedules	2017.10–2018.10
Vallant [30]	UK	CC	IVH PVL	56	22^+0^–23^+6^w	N/A	N/A	2015.01–2021.12
Imren [29]	Netherlands	CC	CP Developmental delay	73	< 32 w/VLBW	24 m	BSID-III	2008–2021
Vaidya [7]	USA	C	ADHD	889	23–27 w	10 y	CSI-4	2002–2004
Culbreath [19]	USA	C	IVH Blind Hearing impairment CP	12,276	ELBW	16–26 m	Bayley III	2011–2017
Tseng [38]	Taiwan	C	Language development delay	3797	VLBW	24 m	Bayley III	2010.01–2015.12

*ADHD* attention-deficit/hyperactivity disorder, *BSID* Bayley Scales of Infant Development, *C* cohort study, *CC* case–control study, *CP* cerebral palsy, *ELBW* extremely low birth weight, *GMFCS* Gross Motor Function Classification System, *IVH* intraventricular haemorrhage, *N/A* not applicable/not available, *NDI* neurodevelopmental impair, *NEC* necrotizing enterocolitis, *PVL* periventricular leukomalacia, *USA* United States Of America, *UK* United Kingdom, *VLBW* very low birth weight, *WPPSI I-III* Wechsler Preschool & Primary Scale of Intelligence

### Brain injury in NEC

**Fig. 2:**
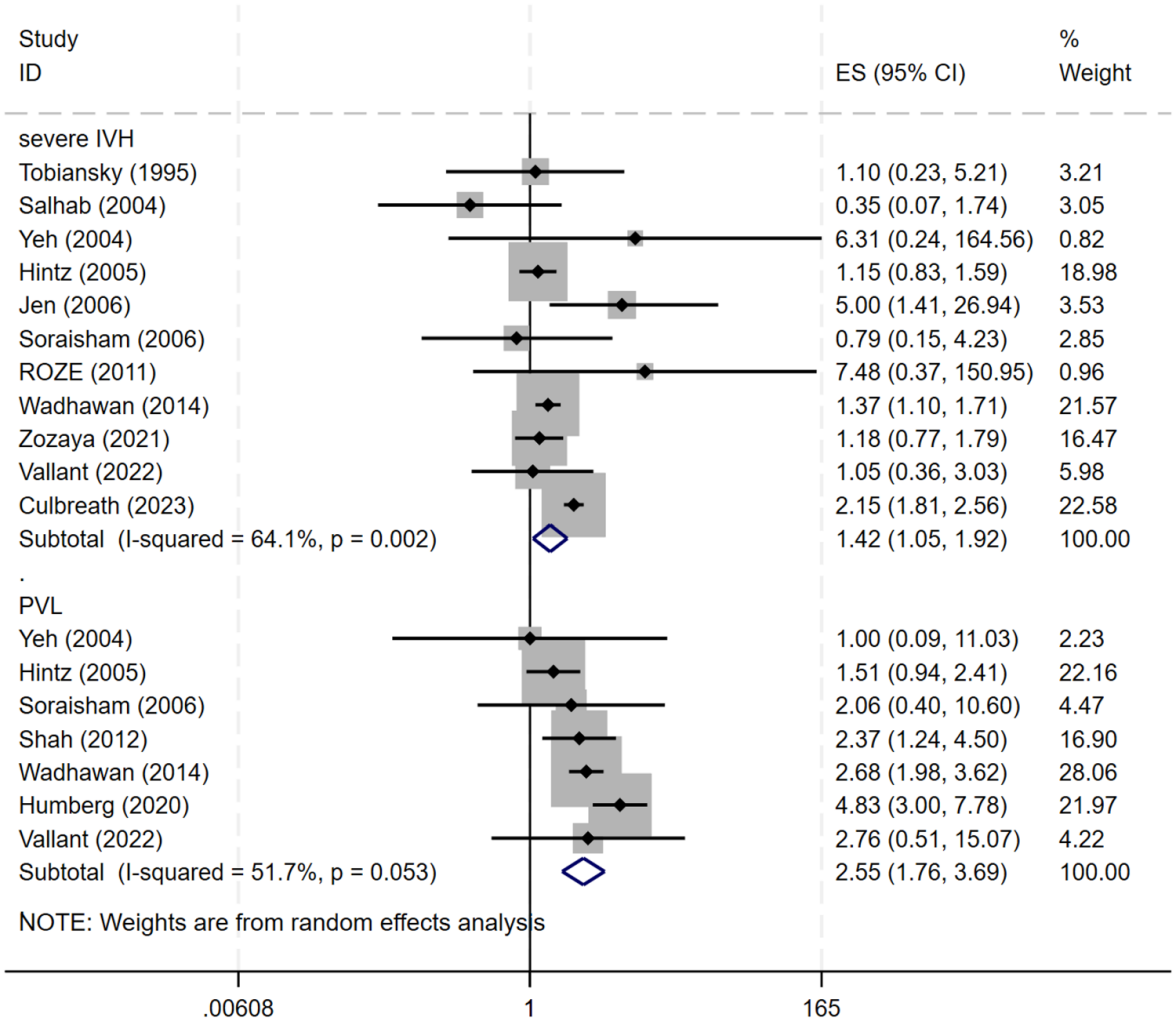
Crude odds ratios expressing the association between severe IVH or PLV and NEC in preterm infants

IVH and PVL are the primary brain injuries that occur in the early postnatal period in preterm infants and are important risk factors for severe long-term neurodevelopmental problems. This study suggests that the risk of severe IVH and PVL in children with NEC is higher than that in those without NEC (OR 1.42, 95% CI 1.06–1.92; OR 2.547, 95% CI 1.76–3.69) (Fig. 2).

### NDI in NEC

**Fig. 3:**
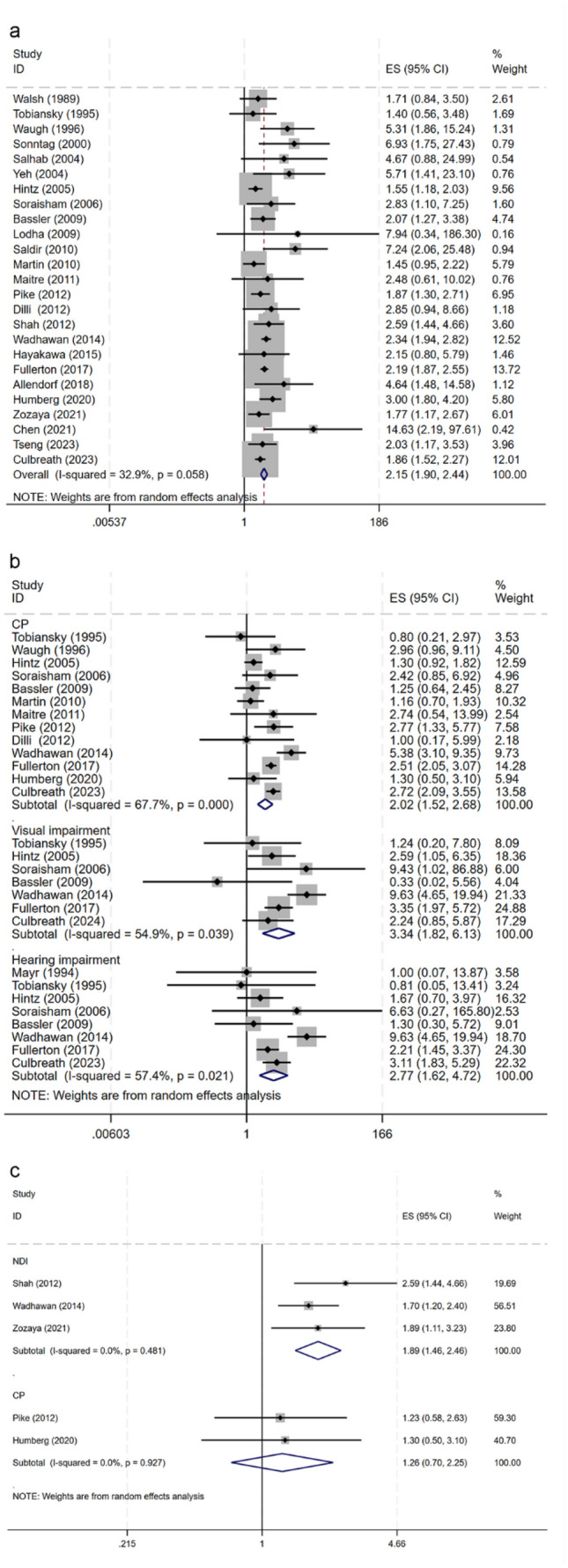
Forest plot of neurodevelopmental impairment (NDI) in preterm infants with necrotising enterocolitis (NEC) compared with none NEC controls. **a** Pooling crude ORs of Overall NDI. **b** Pooling crude ORs of cerebral palsy, visual impairment, and hearing impairment. **c** Pooling adjusted ORs of NDI and cerebral palsy

Our analysis indicated that the risk of NDI in preterm infants was significantly higher in NEC, with a pooled crude OR of 2.15 (95% CI 1.90–2.44) (Fig. 3a) and an adjusted OR of 1.89 (95% CI 1.46–2.46). No statistically significant heterogeneity was found across the studies (*I*^2^ = 32.9%; *P* > 0.05). Pooled unadjusted ORs from studies showed that compared with the non-NEC control group, NEC was significantly associated with CP (OR 2.02, 95% CI 1.52–2.68), visual impairment (OR 3.34, 95% CI 1.82–6.13), and hearing impairment (OR 2.77, 95% CI 1.62–4.72) (Fig. 3b). However, after adjusting for confounders including gestational age, weight, prenatal hormones, and infection, etc. (Supplementary data 2 sTable 2), the adjusted pooled OR (OR 1.26, 95% CI 0.70–2.25) did not suggest a relationship between NEC and the onset of CP (Fig. 3c).

**Fig. 4:**
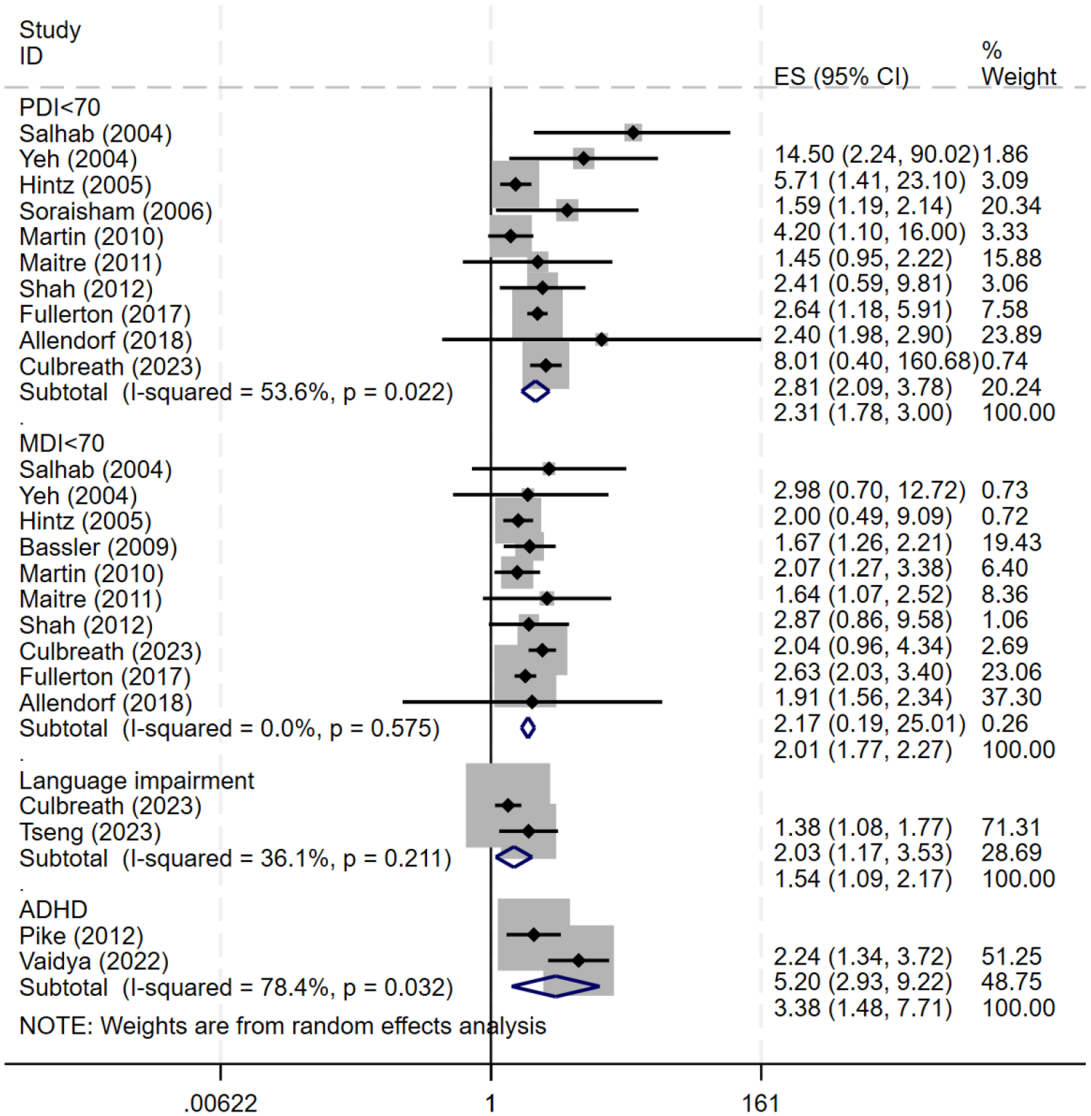
Forest plot comparison of functional impairments in infants with NEC compared with none-NEC controls

Furthermore, the study found the proportion of children with NEC at a corrected age of over 1 year who scored below 70 on the physical development index (PDI) (OR 2.31, 95% CI 1.78–3.00) or mental development index (MDI) (OR 2.01, 95% CI 1.77–2.27) of a developmental assessment, or who had abnormal language assessment scores (OR 1.77, 95% CI 1.11–2.82). This indicates that the risk of motor developmental delay and cognitive and language developmental delay may increase in children with NEC. Although Pike et al. [41] found that ADHD was more common in children with NEC than in controls (15% vs. 8%), the pooled ORs in this study did not suggest a link between ADHD and NEC (OR 1.62, 95% CI 0.79–3.33). However, only three related articles were included in this analysis, exhibiting high heterogeneity (*I*^2^ = 75.2%); further studies are needed to clarify the relationship between ADHD and NEC (Fig. 4).

**Fig. 5:**
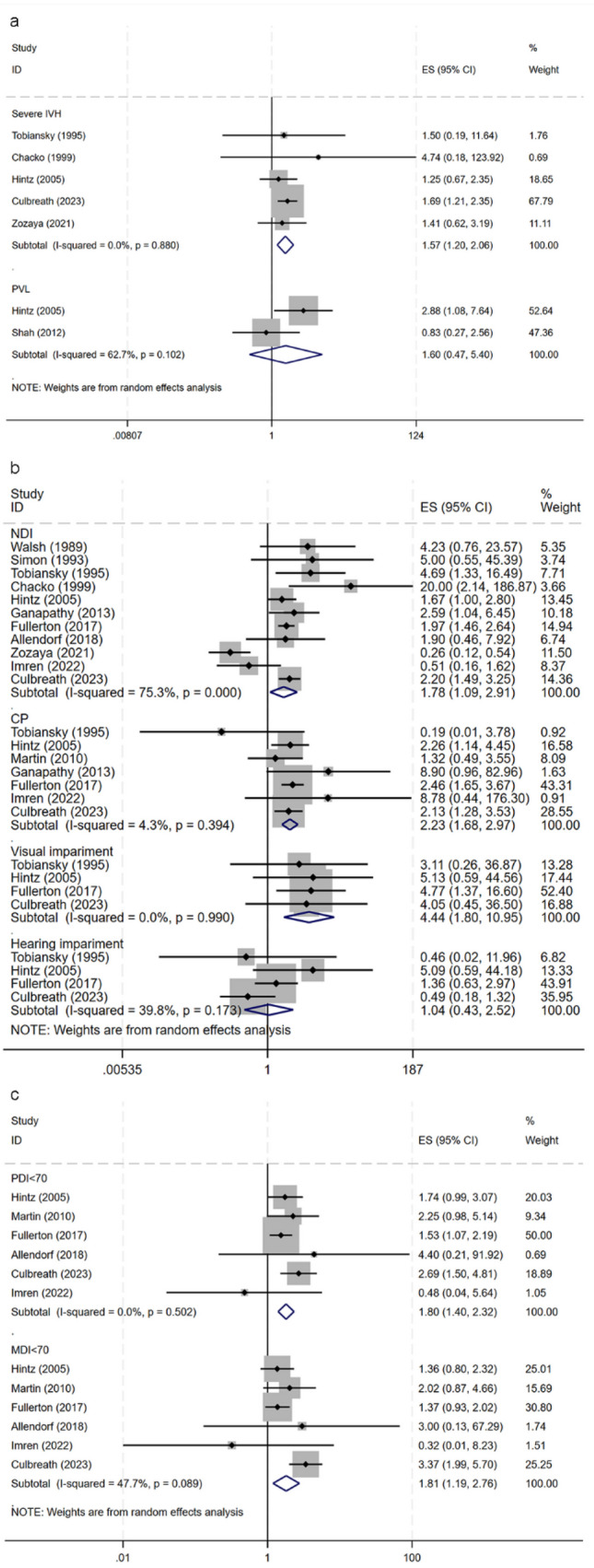
Forest plot of neurodevelopmental impairment (NDI) in preterm infants with surgical NEC compared with medical NEC. **a** Severe IVH and PVL. **b** NDI, cerebral palsy, visual impairment, and hearing impairment. **c** PDI < 70 and MDI < 70

We also studied the effects of NEC severity on brain development. Compared with the surgical NEC group, the risk of severe IVH in the conservatively treated NEC group was higher (OR 1.57, 95% CI 1.20–2.06). However, the risk of PVL was not significantly different between both groups (OR 1.60, 95% CI 0.47–5.40) (Fig. 5a). It is important to note that only two studies were included in the surgical NEC vs. conservative NEC analysis; they exhibited high heterogeneity (*I*^2^ = 62.7%). The risk of NDI in children with surgical NEC was higher than that in the conservative treatment group (OR 1.78, 95% CI 1.09–2.93). Unadjusted pooled ORs indicated that the risk of developing cerebral palsy, visual impairment, and hearing impairment in children who underwent surgery was higher than that in the conservative treatment group (Fig. 5b). Regarding functional development, the risk of motor, intellectual, or language developmental delays was higher in the surgical NEC group than in the non-surgical NEC group (Fig. 5c).

### Sensitivity analyses and publication bias

**Fig. 6:**
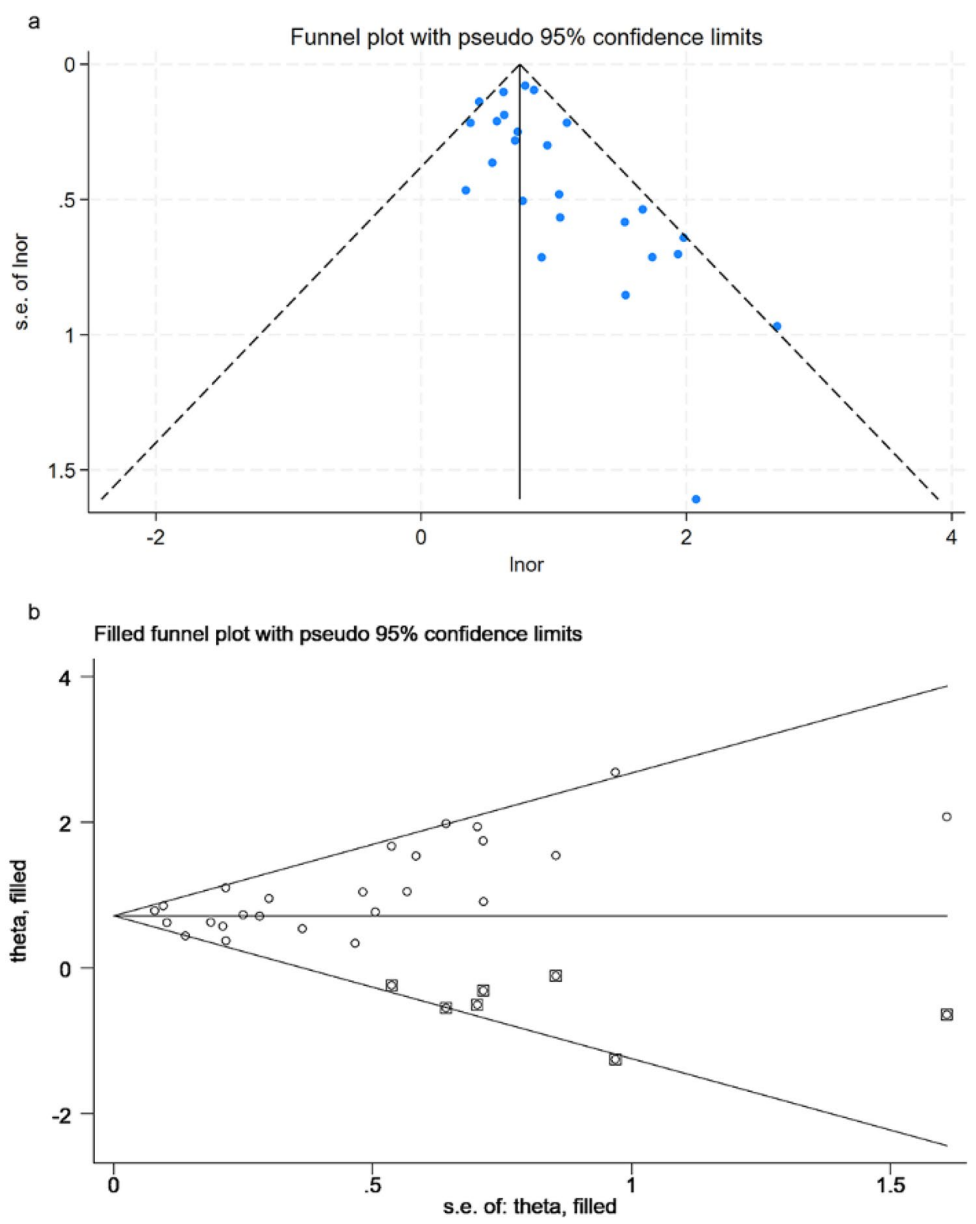
Funnel plots of the association between NDI and NEC in preterm infants. **a** Funnel plot. **b** Funnel plots with trim and fill

The overall pooled result did not vary substantially (Supplementary data 2 sTable 3) after excluding one study in each turn. Visual inspection of the funnel plot indicated a potential publication bias for NDI and NEC, and Begg’s test confirmed statistically significance (*z* = 2.85; *P* = 0.004). We further performed a sensitivity analysis using the trim-and-fill method, which estimated the number of missing studies that may cause funnel plot asymmetry and imputed the hypothetical studies to produce a symmetrical funnel plot (Fig. 6). Although seven theoretically missing trials were incorporated, the analysis still showed a significant association between NDI and NEC.

## Discussion

Our meta-analysis of 33 studies encompassing 60,346 participants indicates that NEC is associated with an increased incidence of NDI, even after adjusting for various confounding factors. In addition, infants requiring surgery for NEC seem to have poorer neurological outcomes than those treated conservatively; however, most of the included studies did not adjust for potential confounders, necessitating further research to solidify this association.

Matei et al. [5] published a systematic review on the neurodevelopmental impairment in necrotizing enterocolitis survivors in 2019. A total of 2403 NEC infants were included, and the incidence of neurodevelopmental impairment was 40%, which was higher in surgical NEC infants than in those with conservative treatment. However, the included literature included full-term and premature infants, and the heterogeneity was high (*I*^2^ = 57%). From 2020 to 2024, there were three studies [19; 21; 28] with large sample reported on the neurodevelopmental injuries in the NEC survivors. In this review, we updated the latest researches, with a larger sample size including a total of 12,409 NEC infants.

Mechanisms underlying NEC-induced brain injury and neurodevelopmental disorders remain unclear. While previously attributed solely to prematurity, studies have shown more severe brain injury in NEC infants compared with non-NEC preemies of equivalent gestational age or those with spontaneous intestinal perforation. This suggests that additional factors beyond prematurity contribute to NEC brain injury [42].

The mechanisms underlying NEC-induced brain injury may be multifactorial. Changes in cerebral haemodynamic may cause brain injury in children with NEC. Children with NEC, especially those who require surgery, often experience hypotension, shock, and acidosis. These can damage cerebrovascular regulatory function, decrease cerebral blood flow, and lead to subsequent hypoxia and ischaemia. The inflammatory response is also considered an important mechanism in the occurrence of NEC-induced brain injury. Animal experiments have shown disrupted brain barrier function in NEC model models, allowing inflammatory blood to enter the brain tissue, activate microglia, trigger neuroinflammation, and ultimately reduce the number of neurons, oligodendrocyte precursors, and neural progenitor cells in the hippocampus, basal ganglia, and cerebral cortex [43, 44]. Beyond the influx of inflammatory factors into the brain, activation of the small intestine’s toll-like receptor (TLR) signalling pathway during NEC leads to the release of high-mobility group box-1 protein, which then enters and activates microglia, resulting in neurofunctional disorders [45]. Additionally, during NEC, enteral CD4 + T enters the brain and releases interferon-γ, activating microglia within the brain and causing myelin loss [46].

Adequate nutrition is a prerequisite for the brain development of preterm infants. Malnutrition during this critical window can lead to reduced brain cells, impaired myelin production, and decreased synapse formation. However, parenteral nutrition (PN) appears to be as efficient as enteral feeding in maintaining adequate nutrition. Children with NEC often receive long-term IV nutrition in the early postnatal period. Studies have shown that parenteral nutrition exceeding 20 days is linked to cognitive impairment in children with NEC aged 2–3 years [47].

This study had several advantages. The large sample size of our study allowed for a much greater possibility of reaching reliable conclusions about the association between NDI and NEC. The positive association between NDI and NEC persisted even after adjusting for confounding factors, demonstrating the high reliability of our findings. Our study also examined the different types of NDI separately. NEC may increase the risk of cerebral palsy in newborns; however, after adjusting for gestational age and birth weight, the combined OR did not suggest a relationship between NEC and cerebral palsy. Therefore, further high-quality studies are needed to clarify this relationship. Our study also suggests that the occurrence of blindness; hearing impairment; and delayed motor, cognitive, and language development during childhood is higher in children with NEC than in those without. However, factors such as gestational age and foetal age may be related to the development of the newborn brain. The possible confounding factors were not adjusted for in the included studies and the related relationships need to be explored further.

The limitations of this study should also be considered. First, most included studies did not adjust for confounding factors affecting child neurodevelopment (e.g., gestational age, birth weight, infection). Second, inconsistency exists in the definition of NDI, and the assessment criteria used are not the same (e.g., while some studies utilised the Bailey scale assessment criterion, others employed the Wechsler scale). Third, most studies were retrospective, with inherent limitations in data control, potentially introducing bias due to incomplete data. Additional well-designed prospective studies reporting adjusted OR are required to confirm our findings.

In conclusion, our study suggests that children with NEC have a higher incidence of brain injury and neurodevelopmental disorders than preterm infants without NEC, and the severity of NDI appears to be related to the degree of intestinal injury. However, the relationship between cerebral palsy and NEC requires further investigation. The risks of blindness, hearing impairment, motor developmental delay, cognitive impairment, and attention deficits seem to be higher in children with NEC than in those without NEC. These findings inform clinical follow-up practices, enabling earlier targeted rehabilitation interventions and potentially improving prognoses. However, most of the included studies did not adjust for confounding factors, and high-quality studies adjusting for factors such as gestational age, birth weight, prenatal hormone use, and infection are needed to definitively assess the NEC-NDI association. Additionally, the mechanisms underlying NDI in children with NEC remain unclear, and effective preventive and treatment methods are lacking. Further research is warranted to address these knowledge gaps.

### Supplementary Information

Below is the link to the electronic supplementary material.

Supplementary file1 (DOCX 31 KB)

Supplementary file2 (XLSX 14 KB)

Supplementary file3 (DOCX 36 KB)

## Data Availability

The data that support the findings of this study are available from the corresponding author upon reasonable request.

## Abbreviations

ADHD: Attention-deficit/hyperactivity disorder
CP: Cerebral palsy
CI: Confidence interval
HRs: Hazard ratios
IVH: Intraventricular haemorrhage
MDI: Mental development index
NDI: Neurodevelopment impairment
NEC: Necrotizing enterocolitis
NOS: Newcastle-Ottawa Scale
ORs: Odds ratios
PDI: Physical development index
PN: Parenteral nutrition
PVL: Periventricular leukomalacia
RRs: Risk ratios
VLBW: Very low birth weight
TLR: Toll-like receptor
