# Supplementary material for: Neurodevelopmental outcomes of preterm with necrotizing enterocolitis: a systematic review and meta-analysis
Source: Eur J Pediatr. 2024 Apr 30;183(8):3147–58. doi: 10.1007/s00431-024-05569-5 (PMC11263237; doi:10.1007/s00431-024-05569-5)
Supplement: Supplementary file 3 — Supplementary file3 (DOCX 36 KB) [file 431_2024_5569_MOESM3_ESM.docx]

**Supplementary Data 2**

**Neurodevelopmental outcomes of premature infants with necrotizing enterocolitis: a systematic review and meta-analysis. Yan Wang, Shunli Liu, Meizhu Lu, Tao Huang, Lan Huang**

**sTable 1 The Newcastle-Ottawa Scale score of included studies**

| **Cohort study** | **Selection of exposed** | **Selection of non-exposed** | **Assess of exposure** | **Outcome** | **Comparability** | **Assess of outcome** | **Long enough of follow up** | **Adequacy of follow up** |
| --- | --- | --- | --- | --- | --- | --- | --- | --- |
| Walsh, 1989 | 1 | 1 | 1 | 1 | 1 | 1 | 1 | 0 |
| Waugh,1996 | 1 | 1 | 1 | 1 | 0 | 1 | 1 | 1 |
| Hintz, 2005 | 1 | 1 | 1 | 1 | 2 | 1 | 1 | 1 |
| Bassler,2009 | 1 | 1 | 1 | 1 | 0 | 1 | 1 | 1 |
| Saldir,2010 | 1 | 0 | 1 | 1 | 0 | 1 | 1 | 1 |
| Pike,2012 | 1 | 1 | 1 | 1 | 1 | 1 | 1 | 1 |
| Martin,2010 | 1 | 1 | 1 | 1 | 1 | 1 | 1 | 1 |
| Shah,2012 | 1 | 1 | 1 | 1 | 1 | 1 | 1 | 1 |
| Wadhawan,2014 | 1 | 1 | 1 | 1 | 0 | 1 | 1 | 1 |
| Fullerton,2017 | 1 | 1 | 1 | 1 | 2 | 1 | 1 | 1 |
| Humberg,2020 | 1 | 1 | 1 | 1 | 2 | 1 | 1 | 1 |
| Zozaya,2021 | 1 | 1 | 1 | 1 | 1 | 1 | 1 | 1 |
| Vaidya，2022 | 1 | 1 | 1 | 1 | 2 | 1 | 1 | 1 |
| Culbreath,2023 | 1 | 1 | 1 | 1 | 2 | 1 | 1 | 1 |
| Tseng,2023 | 1 | 1 | 0 | 1 | 1 | 1 | 1 | 1 |
| **Case control study** | **Definition of cases** | **Representativeness of the Cases** | **Selection of Controls** | **Definition of Controls** | **Comparability of Cases and Control** | **Ascertainment of Exposure** | **Same Method of Ascertainment for Cases and Controls** | **Non-Response Rate** |
| Mayr,1994 | 1 | 1 | 0 | 1 | 0 | 1 | 0 | 0 |
| Tobiansky,1995 | 1 | 1 | 1 | 1 | 1 | 1 | 1 | 0 |
| Chacko,1999 | 1 | 1 | 1 | 0 | 0 | 1 | 1 | 0 |
| Sonntag,2000 | 1 | 1 | 0 | 0 | 0 | 1 | 1 | 0 |
| Salhab,2004 | 1 | 1 | 1 | 1 | 2 | 1 | 1 | 0 |
| Yeh,2004 | 1 | 1 | 1 | 1 | 0 | 1 | 1 | 0 |
| Jen,2006 | 1 | 0 | 0 | 1 | 1 | 1 | 1 | 0 |
| Soraisham, 2006 | 1 | 1 | 1 | 1 | 2 | 1 | 1 | 0 |
| Lodha,2009 | 1 | 0 | 1 | 0 | 0 | 1 | 1 | 0 |
| Simon,1993 | 1 | 1 | 0 | 0 | 0 | 1 | 1 | 0 |
| Maitre,2010 | 1 | 1 | 1 | 1 | 1 | 0 | 0 | 0 |
| Roze，2011 | 1 | 1 | 0 | 1 | 0 | 1 | 1 | 0 |
| Dilli,2012 | 1 | 1 | 1 | 1 | 1 | 1 | 1 | 0 |
| Hayakawa,2015 | 1 | 1 | 1 | 1 | 1 | 1 | 1 | 0 |
| Vallant,2022 | 1 | 1 | 0 | 0 | 0 | 1 | 1 | 0 |
| Imren,2022 | 1 | 1 | 1 | 1 | 0 | 1 | 1 | 1 |
| Chen,2021 | 1 | 1 | 1 | 1 | 0 | 0 | 1 | 0 |
| Allendorf,2018 | 1 | 1 | 1 | 1 | 1 | 1 | 1 | 1 |

**sTable 2 Confounders adjusted in the studies included in the meta-analysis**

| **Outcomes** | **Included**  **studies** | **Confounding factors** |
| --- | --- | --- |
| **NDI** | Shah,2012 | birth weight, race, gender, multiple births, antenatal steroids, surfactant, BPD, sepsis and any IVH |
|  | Wadhawan ,2014 | maternal education, birth weight, male sex, small for gestation (SGA) status, inborn, antenatal antibiotic and corticosteroid therapy, postnatal corticosteroid therapy, bronchopulmonary dysplasia, severe intraventricular hemorrhage, periventricular leukomalacia, late onset sepsis and center |
|  | Zozaya,2021 | gestational age, small for gestational age, prenatal steroids, caesarean section, multiple gestations, and SNAP-II score |
| **Cerebral palsy** | Pike,2012 | maternal age, gestational age at birth, birth weight for gestation ‘Z’ score, mode of birth, antenatal steroid use, sex, multiple birth, ethnicity (white/non-white), housing type (owned/non-owned), PROM, erythromycin and co-amoxiclav, postnatal prescription of antibiotics to the mother |
|  | Humberg,2020 | birth weight, gestational age (per weeks), female gender, ICH ≥ grade 3 and PVL, neurosurgery, European origin, administration of surfactant, BPD, SGA, and maternal education level |
| BPD, bronchopulmonary dysplasia; ICH, intracranial hemorrhage; IVH, intraventricular hemorrhage; NDI, neurodevelopment impairment; PROM, premature rupture of membranes; SGA, small for gestational age; SNAP- II, score for neonatal acute physiologyⅡ | | |

**sTable 3 The sensibility analysis of included studies**

| **Study omitted** | **Estimate** | **95%Confidence Interval** | |
| --- | --- | --- | --- |
| Walsh (1989) | 2.17 | 1.91 | 2.47 |
| Tobiansky (1995) | 2.17 | 1.91 | 2.46 |
| Waugh (1996) | 2.12 | 1.88 | 2.39 |
| Sonntag (2000) | 2.12 | 1.88 | 2.40 |
| Salhab (2004) | 2.14 | 1.89 | 2.43 |
| Yeh (2004) | 2.13 | 1.88 | 2.41 |
| Hintz (2005) | 2.21 | 1.96 | 2.50 |
| Soraisham (2006) | 2.15 | 1.89 | 2.44 |
| Bassler (2009) | 2.16 | 1.90 | 2.47 |
| Lodha (2009) | 2.15 | 1.89 | 2.44 |
| Saldir (2010) | 2.12 | 1.88 | 2.38 |
| Martin (2010) | 2.19 | 1.94 | 2.49 |
| Maitre (2011) | 2.15 | 1.90 | 2.45 |
| Pike (2012) | 2.18 | 1.91 | 2.49 |
| Dilli (2012) | 2.15 | 1.89 | 2.44 |
| Shah (2012) | 2.14 | 1.88 | 2.44 |
| Wadhawan (2014) | 2.14 | 1.86 | 2.46 |
| Hayakawa (2015) | 2.16 | 1.90 | 2.45 |
| Fullerton (2017) | 2.17 | 1.88 | 2.51 |
| Allendorf (2018) | 2.13 | 1.88 | 2.41 |
| Humberg (2020) | 2.10 | 1.85 | 2.38 |
| Zozaya (2021) | 2.18 | 1.91 | 2.49 |
| Chen (2021) | 2.12 | 1.89 | 2.39 |
| Tseng (2023) | 2.16 | 1.90 | 2.47 |
| Culbreath (2023) | 2.20 | 1.92 | 2.53 |
